# A Flexible, Automated, and Basis-Set-Insensitive Domain-Based Charge-Transfer Decomposition for Correlated Wave Functions and Its Application to Inter- and Intramolecular Cases

**DOI:** 10.1021/acs.jpclett.6c01049

**Published:** 2026-05-22

**Authors:** Lena Szczuczko, Julia Szczuczko, Marta Gałyńska, Katharina Boguslawski

**Affiliations:** † Institute of Physics, Faculty of Physics, Astronomy, and Informatics, 317747 Nicolaus Copernicus University in Toruń, Grudzia̧dzka 5, 87-100 Toruń, Poland; ‡ Faculty of Chemistry, 49577 Nicolaus Copernicus University in Toruń, Gagarina 7, 87-100 Toruń, Poland

## Abstract

We present a flexible, automated, and basis-set-insensitive domain-based charge-transfer (CT) decomposition framework that can be combined with any configuration interaction (CI)-type excited-state wave function. Our approach is not based on excited-state densities and allows the excited-state character to be dissected into local and domain-based CT excitations and measures the individual contributions to each excited state. To guarantee a broad applicability, we introduce two domain-accumulation strategies to translate hole-particle substitutions to domain–domain excitations: a strict domain partitioning and a weighted approach suitable for small molecules and a large number of domains. The performance of both schemes is assessed for inter- and intramolecular CT excitations and various basis sets using EOM-CCSD and its simplified counterpart EOM-pCCD+S. Most importantly, the CT character is, to a large extent, basis-set independent, and both domain-accumulation schemes give consistent results. Overall, our framework provides a robust CT analysis and a domain resolution of the excitation character for a variety of computational setups and excited-state models.

## Graphical abstract

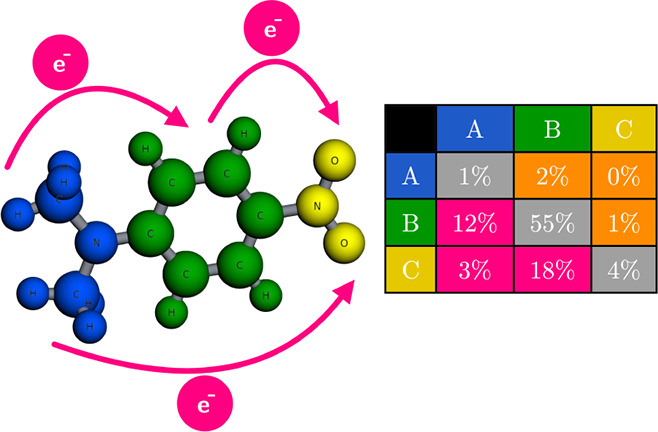

Charge transfer (CT)^1^ plays a central role in key biological processes, such as photosynthesis^2^ and cellular respiration,^3,4^ as well as in the operation of electronic devices, including photovoltaics, organic light-emitting diodes (OLEDs), and chemical sensors.^5–11^ CT may take place within a single molecular entity (intramolecular CT) or between distinct molecular species or across interfaces (intermolecular CT). Depending on the mechanism, it can be photoinduced, arising from electronically excited states or between different conformational states on the same potential energy surface. Despite this diversity, the CT process governs the redistribution of electronic density between molecular fragments and is driven by the presence of electron-rich (donor) and electron-deficient (acceptor) fragments. A detailed understanding of the spatial distribution of charge across those molecular domains is of particular interest. Especially in terms of electronic excitations, it is essential to characterize the nature of the excited state, particularly whether an excitation is local or involves CT between fragments. However, the description of electronic excitations depends critically on the theoretical framework employed, and the methodology used to analyze excited states must be chosen accordingly.

Understanding the spatial character of electronic excitations, specifically, whether they are local or involve CT, constitutes an important aspect of excited-state chemistry. Excited-state analysis methods can be broadly categorized by how electronic excitations are represented. In linear-response approaches, such as time-dependent density functional theory (TD-DFT)^12^ or linear response coupled cluster (LR-CC),^13,14^ excited states are treated as perturbations of the ground state. In this formalism, the transition density matrix (TDM), which couples the ground and excited states, arises naturally as a central quantity.^15^ Together with density difference, they provide a compact representation of electronic excitations in terms of electron–hole transitions and enable the construction of several descriptors such as natural transition orbitals, attachment/detachment analysis, fragment-based analysis, exciton size, average position of hole and particle, charge transfer distance, or hole/electron delocalization, to name a few.^15–24^ This type of analysis is available in dedicated tools such as TheoDORE,^25^ Multiwfn,^26^ VALET,^27^ or implemented into quantum chemistry packages such as Gaussian,^28^ Orca,^29,30^ Q-Chem,^31^ OpenMolcas,^32^ or Veloxchem.^33,34^ In contrast, equation of motion (EOM) for excitation energies^35–37^ and related formalisms^38–42^ describe excited states as explicit many-electron states built from the ground-state reference via excitation operators. In these approaches, the excited-state wave function is represented in terms of Configuration Interaction (CI) expansion coefficients, which directly encode the contributions of individual orbital transitions. While transition density matrices can also be derived within the EOM framework, their construction requires additional effort, as both left and right eigenstates must be computed and combined.^36^ Therefore, for this particular case, an alternative approach to excited-state analysis is desirable, specifically, one that does not rely on excited-state densities.

While excited states are rigorously defined through wave function expansions, interpreting *where* electron density moves remains nontrivial. Traditional approaches, such as orbital inspection or density difference plots or natural transition orbitals, often provide only qualitative insight and may depend strongly on the chosen representation and individual interpretation. Domain-based approaches address this limitation by introducing an explicit spatial partitioning of the system. Recently, we introduced a domain-based CT analysis for electronically excited states computed with the simple EOM pair coupled cluster doubles (pCCD) with singles correction (EOM-pCCD+S) method^43–48^ and applied it to donor–bridge–acceptor dyes, designed as prototypical systems for dye-sensitized solar cell applications.^49–51^ Our original approach was based on a manual, tedious, and time-consuming evaluation of excited-state contributions and restricted to single excitations, which severely limits its applicability. Here, we generalize this approach and present a flexible, automated scheme to dissect the character of excited states into local and interdomain excitations (or CT-type excitations) for any correlated CI-type wave function expansion and different choices for domain accumulation. Importantly, the framework applies to any correlated CI-type excited-state method that does not require explicit evaluation of left eigenvectors (in the case of EOM-type methods) or excited-state densities. Nonetheless, our methodology can be generalized, and the CT analysis can be performed using the right eigenvector only (as discussed in this work), the left eigenvector in addition to the right ones, or an approximate left eigenvector constructed from the right one.^52^ We test our approach for both intra- and intermolecular systems and scrutinize its basis set dependence. Specifically, we probe our analysis for EOM coupled cluster (CC) singles and doubles (CCSD) and compare the directed domain-based CT character to other EOM-CCSD data present in the literature, for both inter- and intramolecular CT excitations. We should stress that the aim of this work is to provide an alternative to density-based partition schemes of excited states that can be applied for any CI-type excited state wave function. We further aim at an efficient evaluation of our CT matrix and the derived CT indices. Thus, we exploit the right eigenvectors only. Although being an approximation within the EOM-CC formalism, our numerical data support this shortcut. Note, however, that the proposed methodology is not restricted to this approximation.

In our flexible, automated, domain-based CT framework, the molecule is divided into chemically relevant regions (domains), and excitations are analyzed in terms of electron transfer between these regions (see also Figure 1 for a schematic representation). Our framework is valid for any CI-type excited-state wave function, where the excited state of interest |Ψ_*k*_⟩ is parametrized as

1 $$\left|\Psi_{k}\right\rangle =\underset{\mu =0,S,D,...}{\sum }c_{\mu }{\mathrm{\tau ̂}}_{\mu }\left|\Psi_{0}\right\rangle$$

where the sum runs over all possible single, double, etc. excitations and τ̂_μ_ represents an excitation operator (supplemented by the identity operator τ̂_0_) that promotes electrons from the occupied to the virtual orbital space (related to some reference determinant), while |Ψ_0_⟩ encodes the ground-state wave function. In the following, we will benchmark our domain-based CT framework for excited states modeled within the EOM-CCSD model. Thus, we will restrict the above ansatz to single and double excitations and derive the CT measures within this excitation manifold. However, our approach can be generalized to higher-order excitations. Restricting the ansatz to single and double excitations, eq 1 can be cast into

2 $$\left|\Psi_{k}^{SD}\right\rangle =\left(c_{0}{\mathrm{\tau ̂}}_{0}+\overset{\mathrm{occ}}{\underset{i}{\sum }}\overset{\mathrm{virt}}{\underset{a}{\sum }}c_{i}^{a}{\mathrm{\tau ̂}}_{ai}+\overset{\mathrm{occ}}{\underset{i,j}{\sum }}\overset{\mathrm{virt}}{\underset{a,b}{\sum }}c_{ij}^{ab}{\mathrm{\tau ̂}}_{abij}\right)\left|\Psi_{0}\right\rangle$$

where τ̂_*ai*_ encodes single excitations (from an occupied orbital *i* to a virtual orbital *a*) and τ̂_*abij*_ creates a doubly excited configuration. We should note that we have dropped all prefactors in the above equation as they will depend on the chosen excited-state parametrization (spin-free vs spin-integrated formalisms).

**Figure 1:**
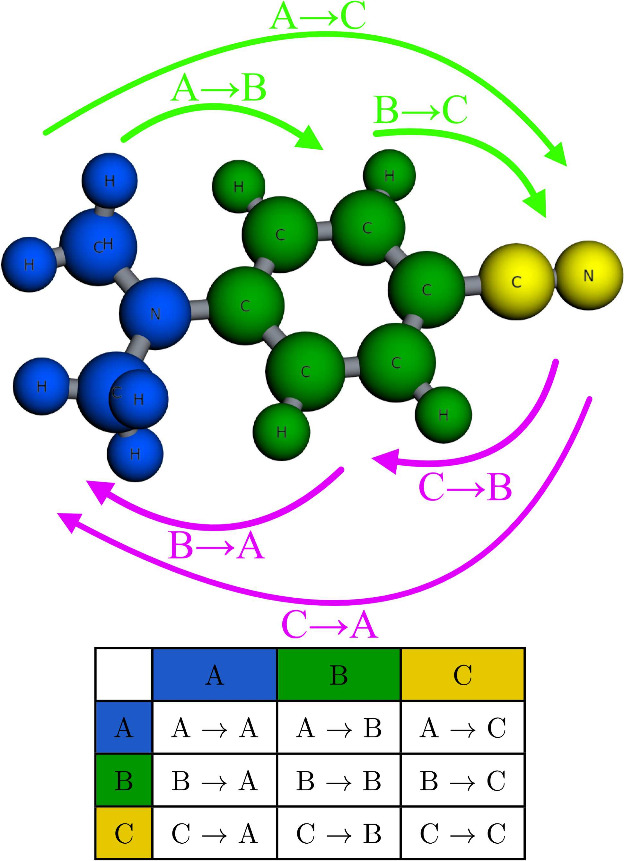
Schematic representation of a three-domain CT analysis across a donor–bridge–acceptor system. The corresponding domain–domain excitation matrix is shown below, comprising the individual initial-to-final domain excitations. The donor (A, blue), bridge (B, green), and acceptor (C, yellow) define forward (green arrows) and backward (magenta arrows) charge transfer pathways. The net directed CT is obtained by summing all forward contributions (A → B, B → C, A → C) and subtracting the corresponding reverse processes (A ← B, B ← C, A ← C).

In CC theory, the correlated many-electron wave function is expressed by an exponential ansatz acting on a single-reference determinant |Φ_0_⟩, usually a Hartree–Fock (HF) determinant. In this work, we consider two restrictions of the general CC ansatz, namely, we restrict our analysis to a CCSD ground-state reference function, and we focus on spin-free, singlet excitations solely. The corresponding CCSD ansatz,

3 $$\left|\Psi_{\mathrm{CCSD}}\right\rangle =e^{{\mathrm{T̂}}_{1}+{\mathrm{T̂}}_{2}}\left|\Phi_{0}\right\rangle$$

then includes spin-free single (*T̂*_1_) and double (*T̂*_2_) excitation operators, which are defined through the singlet excitation operator *E*_*i*_^*a*^ = *â*_*a*_^†^ *â*_*i*_ + *â*_a̅_^†^ *â*_*i̅*_ as follows

4 $${\mathrm{T̂}}_{1}=\overset{\mathrm{occ}}{\underset{i}{\sum }}\overset{\mathrm{virt}}{\underset{a}{\sum }}t_{i}^{a}E_{i}^{a}$$

and

5 $${\mathrm{T̂}}_{2}=\frac{1}{2}\overset{\mathrm{occ}}{\underset{i,j}{\sum }}\overset{\mathrm{virt}}{\underset{a,b}{\sum }}t_{ij}^{ab}E_{i}^{a}E_{j}^{b}$$

where *t*_*i*_^*a*^ and *t*_*ij*_^*ab*^ denote single and double excitation amplitudes, respectively, and *â*_*a*_^†^ and *â*_*i*_ are the creation and annihilation operators for α (*a*) and β (*a̅*) spin. In this spin-free picture, the excitation operator τ̂ in eq 2 can be identified as τ̂_*ai*_ = *E*_*i*_^*a*^ and τ̂_*abij*_ = *E*_*i*_^*a*^ *E*_*j*_^*b*^, respectively. Despite these restrictions, our analysis remains valid for the spin-integrated case. Assuming spin-free excitations allows for a compressed representation of excited states, which reduces the overall storage of excitation amplitudes and thus speeds up the underlying analysis.

To resolve an excited state into domain contributions, we introduce a domain–domain excitation matrix, which comprises both local (or intradomain) and interdomain excitations (see Figure 1 for a three-domain example). Specifically, this domain–domain excitation matrix is constructed by accumulating CI contributions according to the domain character of the participating orbitals (that is, deduced from hole-particle excitations). For a given orbital *p* and domain *D*, we define a scheme-dependent domain factor

6 $$\Omega_{D}\left(p\right)=\{\begin{array}{lll} 1 & \mathrm{if}D=\arg\underset{D^{\prime }}{\max}\underset{\mu \in D^{\prime }}{\sum }{\left|C_{\mu p}\right|}^{2};\mathrm{otherwise},0 & \left(\mathrm{discrete}\mathrm{or}\mathrm{hard}\mathrm{scheme}\right)\\ \underset{\mu \in D}{\sum }{\left|C_{\mu p}\right|}^{2} & & \left(\mathrm{weighted}\mathrm{scheme}\right) \end{array}$$

where *C*_*μp*_ are the orthonormalized atomic orbital (AO) expansion coefficients of molecular orbital *p*, and the sum runs over all atomic basis functions μ belonging to domain *D*. Note that *C*_*μp*_ are obtained from the nonorthonormal expansion coefficients through *S*_*μ ν*_^1/2^ C̃_*νp*_, where *S* is the overlap matrix of the AO basis. For the hard scheme, Ω_*D*_(*p*) takes only the discrete values 0 or 1, indicating whether orbital *p* is assigned to domain *D* (1) or not (0). In contrast, in the weighted scheme Ω_*D*_(*p*) assumes any value between 0 and 1 and reflects how strongly orbital *p* is localized on domain *D*, as determined by the AO–MO expansion coefficients. This distinction clarifies that the hard scheme enforces a strict domain assignment, whereas the weighted scheme provides a continuous measure of domain contribution. In the context of atom-centered basis sets, a domain is, hence, defined as a collection of atoms (see also Figure 1). Using this definition, the domain-based excitation matrix contribution for single excitations is given by

7 $$M_{D_{h}D_{p}}^{\left(S\right)}=\overset{\mathrm{occ}}{\underset{i}{\sum }}\overset{\mathrm{virt}}{\underset{a}{\sum }}{\left|c_{i}^{a}\right|}^{2}\Omega_{D_{h}}\left(i\right)\Omega_{D_{p}}\left(a\right)$$

where *i* and *a* denote occupied and virtual orbitals, respectively, and *c*_*i*_^*a*^ are the corresponding CI amplitudes of the excited state in question. Note that *M*_*D*_*h*_ *D*_*p*__ contains the *D*_*h*_ → *D*_*p*_ domain excitation element, where *D*_*h*_ is the initial domain (hole states) and *D*_*p*_ the final domain (particle states). Double excitations are implemented within a one-electron channel concept, in which each excitation (*i*,*j*) → (*a*,*b*) is decomposed into two contributions, yielding (in the spin-free formulation)

8 $$M_{D_{h}D_{p}}^{\left(D\right)}=\frac{1}{2}\overset{\mathrm{occ}}{\underset{i,j}{\sum }}\overset{\mathrm{virt}}{\underset{a,b}{\sum }}{\left|c_{ij}^{ab}\right|}^{2}\left[\Omega_{D_{h}}\left(i\right)\Omega_{D_{p}}\left(a\right)+\Omega_{D_{h}}\left(j\right)\Omega_{D_{p}}\left(b\right)\right]$$

where *c*_*ij*_^*ab*^ denote double-excitation amplitudes and (*i*,*j*) and (*a*,*b*) label occupied and virtual orbitals involved in the excitation. We should stress that, in the spin-free case, eq 8 yields eq 7 for the case of electron-pair excitations where *i* = *j* and *a* = *b*. Note that, in the spin-free case, electron-pair excitations are composite single excitations which are given by the *c*_*ii*_^*aa*^ excited-state vector contribution. We emphasize once more that all expressions and operator relations used in this work are formulated within the spin-free framework.

The resulting domain-based excitation matrix provides a compact representation of charge redistribution between domains. Diagonal elements correspond to local excitations, while off-diagonal elements quantify interdomain charge transfer. As illustrated in Figure 1, these contributions can be interpreted as directed CT pathways between domains. We should note that the sum over all domain-based excitation matrix elements is the same for both scheme-dependent domain factors. The outlined framework provides a general and method-independent approach to analyze the CT character in excited states represented by CI-type wave functions. Its practical realization is implemented in the Domain Assignment and Interface Solution in pYthon (DAISpY) package,^53,54^ which automates domain construction, orbital assignment, and the accumulation of domain–domain excitation matrices for selected excited states.

To quantify the directionality of the charge flow in, for instance, a donor–bridge–acceptor system (see Figure 1), we introduce a directed charge transfer (dCT) measure. This quantity accounts for all possible pairwise CT contributions between the donor (A), bridge (B), and acceptor (C), while explicitly differentiating between forward (donor-to-bridge-to-acceptor) and backward (acceptor-to-bridge-to-donor) flow. Positive contributions correspond to CT along the forward direction, whereas negative contributions capture CT in the opposite direction. The resulting expression therefore provides a compact measure of the overall CT across the system. For a collection of *q* domains {*D*_*q*_}, the dCT from *D*_1_ to *D*_2_ to *D*_3_ etc. can be evaluated from

9 $$\mathrm{dCT}=\overset{D_{1}\to ...\to D_{q}}{\underset{D_{h},D_{p}}{\sum }}\left[D_{h}\to D_{p}-D_{h}\leftarrow D_{p}\right]$$

For the donor–bridge–acceptor pathway described above and visualized in Figure 1, we explicitly obtain

10 dCT(A→B→C)=[(A→B)+(B→C)+(A→C)]−[(A←B)+(B←C)+(A←C)]

This expression corresponds to taking the difference between the upper and lower triangular parts of the CT matrix, excluding the diagonal. Moreover, the donor, acceptor, and bridge labels used throughout this work are not assigned a priori. Instead, the roles of individual domains are determined a posteriori from the computed dCT flow. In other words, the domain classification emerges naturally from the analysis itself and the physicochemical process under consideration, rather than being imposed beforehand, ensuring that the interpretation of charge redistribution is fully consistent with the underlying data.

Finally, to obtain unambiguous domain-to-domain excitations, it is essential that the automated domain decomposition can be performed unequivocally, where hole or particle indices are uniquely associated with domain indices. If our domain decomposition is performed on delocalized canonical orbitals, the corresponding domain accumulation might yield smeared-out weighting factors, deforming a domain-based CT analysis. For that purpose, all wave function optimizations are performed within a localized molecular orbital (MO) basis. In principle, we could employ any localized MOs, such as Pipek–Mezey^55,56^ ones or others.^57,58^ However, to obtain strongly localized virtual orbitals, we exploit pCCD-optimized^43–45,48^ MOs. Unlike the standard CC Doubles (CCD) approach, which includes all possible double excitations, pCCD employs a simplified ansatz that selectively accounts only for paired electron excitations. The pCCD wave function is formulated using an exponential ansatz,^45,48,59^

11 $$\left|\Psi_{\mathrm{pCCD}}\right\rangle =e^{\sum_{i}^{\mathrm{occ}}\sum_{a}^{\mathrm{virt}}t_{i\mathrm{i̅}}^{a\mathrm{a̅}}{â}_{a}^{†}{â}_{\mathrm{a̅}}^{†}{â}_{\mathrm{i̅}}{â}_{i}}\left|\Phi_{0}\right\rangle =e^{{\mathrm{T̂}}_{2}^{\mathrm{pCCD}}}\left|\Phi_{0}\right\rangle$$

where the MOs used to construct the reference determinant are typically optimized using a variational orbital optimization procedure.^45,59,60^ The optimal set of orbitals results in a vanishing pCCD orbital gradient (in spatial orbitals),

12 gpq=⟨Φ0|e−T̂2pCCD[(Êpq−Êqp),Ĥ]eT̂2pCCD|Φ0⟩+∑i,aλii̅aa̅(⟨Φii̅aa̅|e−T̂2pCCD[(Êpq−Êqp),Ĥ]eT̂2pCCD|Φ0⟩)=0⁣∀p>q

where λ_*ii̅*_^*aa̅*^ are the Lagrange multipliers obtained from the pCCD Λ equations, *Ĥ* represents the molecular Hamiltonian, and we explicitly use α- and β-spin labels to stress the pair nature of the ansatz. The bra state ⟨Φ_*ii̅*_^*aa̅*^| corresponds to a pair-excited Slater determinant defined as |Φ_*ii̅*_^*aa̅*^⟩ = *â*_*a*_^†^ *â*_*a̅*_^†^ *â*_*i̅*_ *â*_*i*_ |Φ_0_ ⟩.^45,59,60^ The resulting pCCD-optimized natural orbitals (that is, those with a vanishing orbital gradient) are strongly localized. We should stress that in this work we consider pCCD mainly as a means to provide localized orbitals for both the occupied and virtual subspaces, respectively.

We first assess the CT analysis based on our automated domain-based framework discussed above for a set of 10 two-component molecular systems presented in Figure 2a. Table 1 shows the DAISpY CT analysis results for EOM-CCSD compared to EOM/LR-CCSD reference values from a previous study.^61^ The corresponding comparison using EOM-pCCD+S is provided in the Supporting Information (SI). While our CT character is reported as the one-directional CT between the donor and acceptor fragments, i.e., the nonlocal component of the charge redistribution or the CT matrix element for the A → B transition, and displayed in fractional values, the previous study defines their ω_*CT*_ as the weight of configurations with charges separated on different fragments. In both cases, the excitation character is more local when the corresponding value is close to 0, whereas values approaching 1 indicate a stronger CT character.

**Figure 2:**
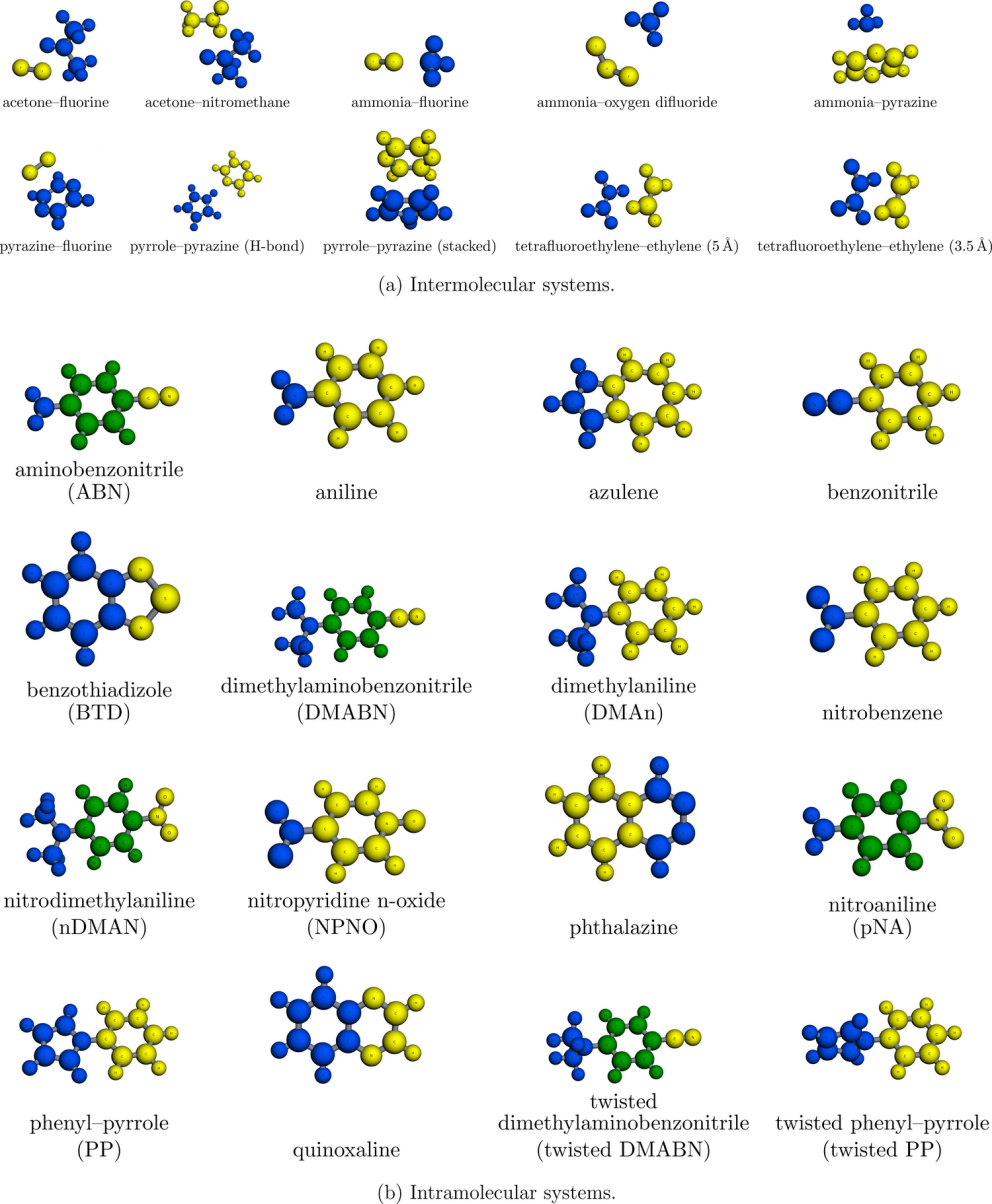
All investigated systems exhibiting CT. Colors indicate molecular roles: blue for the donor, green for the bridge, and yellow for the acceptor domain. The visualizations were generated using 3Dmol via DAISpY’s graphical user interface.

**Table 1:** Intermolecular CT Character (All CT Values Are of 1 → 2 Character, i.e., from the First Fragment Listed in the Molecular Name to the Second) and Excitation Energies (EE in eV) Calculated with cc-pVDZ (DZ) and cc-pVTZ (TZ) Basis Sets Using EOM-CCSD­(pCCD) and Reference EOM/LR-CCSD­(HF) Data^a^

		CCSD(HF)^61^		CCSD(pCCD)	
molecule	basis set	EE	ω_*CT*_	EE	CT
acetone–fluorine	DZ	6.28	0.96	6.33	0.94
	TZ			6.02	0.93
acetone–nitromethane	DZ	6.75	0.80	6.84	0.69
	TZ			6.64	0.68
ammonia–fluorine	DZ	6.90	0.76	6.87	0.79
	TZ			6.63	0.79
ammonia–oxygendifluoride	DZ	7.33	0.86	7.37	0.84
	TZ			7.33	0.61
ammonia–pyrazine	DZ	7.93	0.63	7.95	0.61
	TZ			7.81	0.75
pyrazine–fluorine	DZ	6.73	0.64	6.74	0.64
		6.77	0.98	6.77	0.94
	TZ			6.28	0.90
				6.37	0.95
pyrrole–pyrazine (H-bond)	DZ	5.60	1.00	5.64	0.96
		6.32	0.97	6.32	0.92
		6.47	0.99	6.51	0.95
pyrrole–pyrazine (stacked)	DZ	5.68	0.84	5.69	0.81
		6.22	0.66	6.24	0.62
		6.52	0.61	6.51	0.58
tetrafluoroethylene–ethylene (5 Å)	DZ	10.87	0.99	10.87	0.97
	TZ			10.54	0.94
tetrafluoroethylene–ethylene (3.5 Å)	DZ	9.05	0.34	9.08	0.36
	TZ			8.66	0.35

a Missing values have not been computed. HF, indicates that canonical Hartree–Fock orbitals are used to construct the reference determinant. pCCD, indicates a reference determinant constructed with pCCD-optimized orbitals. The corresponding comparison with EOM-pCCD+S data is available in Table S4 of the SI.

For the intermolecular CT analysis, Table 1 reports the values obtained using the hard partitioning scheme, while a weighted CT_*w*_ analysis is provided in the SI for the cc-pVTZ basis set. Since the systems considered here consist of two separated molecular fragments, both partitioning schemes yield very similar results. For this reason, the comparison is limited to the cc-pVTZ basis set, and only the hard CT values are discussed in the main text. The EOM-CCSD states calculated using pCCD orbitals (labeled as EOM-CCSD­(pCCD)), identified by DAISpY as CT states, show excellent agreement with the reference EOM/LR-CCSD results computed using canonical HF orbitals (EOM/LR-CCSD­(HF)). These findings confirm that the CT characterization is not tied to a particular orbital representation of the EOM-CCSD states. However, the analysis framework itself presupposes a localized orbital basis, for which the pCCD-optimized orbitals provide a consistent and reliable choice. The excitation energy differences between EOM-CCSD­(pCCD) and EOM/LR-CCSD­(HF) results are typically within chemical accuracy (around 0.04 eV; a larger error of 0.1 eV was found for acetone–nitromethane), confirming that EOM-CCSD is largely independent of the choice of orbitals in the reference wave function.

As expected, the excitation energies obtained with EOM-pCCD+S (see the SI) are generally higher than their EOM-CCSD equivalents. The differences between them are around 2 and 6 eV. Overall, the CT values predicted with EOM-pCCD+S are systematically lower than those obtained with EOM-CCSD and do not quantitatively coincide. However, the underlying CT character, such as the direction and extent of CT, is qualitatively preserved. Increasing the basis set size from DZ to TZ quality, overall yields similar CT weights (exceptions can be found for ammonia–oxygendifluoride, pyrazine–fluorine, and pyrazine–fluorine). This demonstrates that the DAISpY-based CT analysis is more basis-set insensitive (compared to excitation energies) and that EOM-pCCD+S provides a cheap, but reliable alternative to EOM-CCSD for extracting CT indices, offering comparable qualitative performance at a significantly lower computational cost.

As a second test set, we benchmark our DAISpY-driven flexible, automated domain-based CT framework for the intramolecular case comprising 16 molecules shown in Figure 2b. In this particular case, the investigated systems were divided into donor and acceptor domains, and in some cases, a bridge domain was included between them. The CT states were selected based on reference data from a previous study.^62^ Our results obtained with EOM-CCSD using the cc-pVTZ basis set are summarized in Table 2 and Figure 3c, while the basis set dependence for EOM-CCSD (for both hard dCT and weighted dCT_*w*_) is illustrated in Figure 3a,b. The corresponding analysis for EOM-pCCD+S, including both basis set effects and deviations from EOM-CCSD, is shown in Table S4 and Figure S2 in the SI. We should stress that, for all methods and basis sets, we consider the dCT values only (see eq 10), which quantifies the net charge flow from the donor to the bridge (if present) to the acceptor domain.

**Table 2:** Intramolecular Hard (dCT) and Weighted (dCT_*w*_) dCT Character and Excitation Energies (EE in eV) Calculated with the cc-pVTZ Basis Set Using EOM-CCSD^a^

molecule	dCT char.	EE	dCT	dCT_*w*_
ABN	++	5.41	0.29	0.23
aniline	++	5.99	0.23	0.18
benzonitrile	++	7.33	0.27	0.27
DMAn 1	++	4.66	0.21	0.18
DMAn 2	++	5.68	0.36	0.29
nitrobenzene	+++	5.77	0.53	0.48
NPNO	++	4.45	0.31	0.29
azulene 1	++	4.03	0.41	0.20
azulene 2	+	4.83	0.24	0.10
BTD	++	4.67	0.34	0.19
DMABN	++	5.10	0.40	0.35
pNA	+++	4.81	0.50	0.44
nDMAN	+++	4.53	0.62	0.56
PP 1	+++	5.87	0.42	0.41
PP 2	++++	6.56	0.75	0.74
phtalazine 1	++	4.28	0.09	0.17
phtalazine 2	++	4.64	0.30	0.34
quinoxaline 1	+++	5.04	0.59	0.54
quinoxaline 2	++	5.98	0.34	0.30
twisted DMABN 1	++++	4.42	0.84	0.77
twisted DMABN 2	++++	5.19	0.83	0.76
twisted PP 1	++++	6.13	0.87	0.85
twisted PP 2	+++	6.22	0.62	0.62
twisted PP 3	++++	6.33	0.80	0.75
ME (DZ)	–	–	0.01	0.02
SD (DZ)	–	–	0.06	0.07
ME (ADZ)	–	–	–0.03	–0.02
SD (ADZ)	–	–	0.10	0.10
ME (ATZ)	–	–	–0.02	–0.04
SD (ATZ)	–	–	0.06	0.06

a The dCT is evaluated according to eq 10. For systems with multiple CT states, numerical labels denote the first, second, and subsequent states, ordered by excitation energy. The qualitative dCT character (dCT char.) is assigned based on the weighted EOM-CCSD dCT_*w*_ values: weak (+) for |dCT_*w*_| ≤ 0.10, moderate (++) for 0.10 < |dCT_*w*_| ≤ 0.35, strong (+++) for 0.35 < |dCT_*w*_| ≤ 0.7, and (mostly) pure (++++) for 0.7 < |dCT_*w*_| ≤ 1.0. Mean error (ME) and standard deviation (SD) are also provided for the cc-pVDZ (DZ), aug-cc-pVDZ (ADZ), and aug-cc-pVTZ (ATZ) basis sets, using the cc-pVTZ results as reference values. The distribution of these statistics is illustrated by the violin plots shown in Figure 3. The parallel analysis for EOM-pCCD+S data is available in Table S5 and Figure S2 the SI.

**Figure 3:**
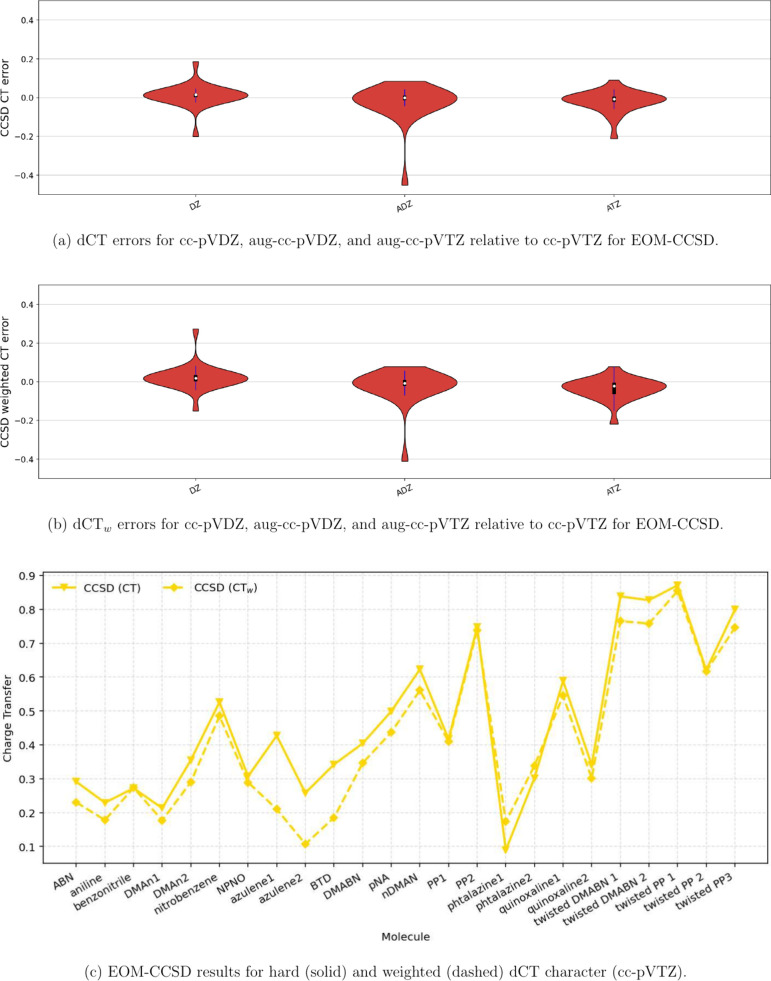
CT analysis of excited states for EOM-CCSD. The upper panels show violin plots of dCT and dCT_*w*_ errors for different basis sets relative to cc-pVTZ. The lower panel compares EOM-CCSD results for strict and weighted dCT character. For systems with multiple CT states, numerical labels denote the first, second, and subsequent states, ordered by excitation energy.

Overall, the flexible, automated domain-based CT framework predicts a wide range of CT states, from weak charge-transfer character (e.g., aniline, indicated by a “+” in the table) to strongly charge-separated states (e.g., nitrobenzene, pNA, or nDMAN, indicated by a “+++” in the table) to almost pure CT states (e.g., PP and twisted PP and DMABN systems, indicated by a “++++” in the table). As expected, twisted systems exhibit a strong charge separation and feature excited states of almost pure dCT character. Slightly smaller, albeit still significant dCT values are obtained for donor–bridge–acceptor assemblies, such as pNA or nDMAN. The weakest dCT values are observed for 2-domain molecules comprising a donor–ring configuration, followed by undoped, C-only systems (like azulene).

Furthermore, both the hard and weighted CT values are rather basis-set-insensitive (see Figure 3), as the error distributions for both descriptors are closely centered around zero for all basis sets considered (with a standard deviation at around 6% for larger basis sets). Specifically, for the hard CT flavor, the spread of the distribution remains relatively uniform across the cc-pVDZ, aug-cc-pVDZ, and aug-cc-pVTZ basis set, indicating a stable behavior with respect to basis set enlargement. In contrast, the weighted CT_*w*_ exhibits slightly broader distributions for smaller basis sets, particularly for aug-cc-pVDZ, where a few outliers are visible. Nevertheless, these deviations remain modest and do not affect the overall conclusion that basis set effects are minor. Importantly, the absence of larger shifts in the median values confirms that no significant bias is introduced by the choice of basis set. This robustness is essential for practical applications, as it allows the use of smaller basis sets without compromising the qualitative interpretation of the CT character.

Furthermore, comparing the hard dCT and weighted dCT_*w*_ measures shown in Figure 3 reveals a rather consistent description between both domain accumulation recipes across systems of varying size and complexity. However, we recommend the weighted CT_*w*_ framework, especially if the molecules to be partitioned are small and feature domains that are connected by more than one interatomic connection. In particular, CT_*w*_ is less sensitive to the partitioning into domains and remains additive when multiple charge-transfer pathways are present. This becomes particularly evident for systems such as phthalazine, where different domain partitions (e.g., two versus three domains; see also Table S2 in the SI) can be considered. In this case, the weighted CT_*w*_ measure provides a consistent and additive description, whereas the hard CT definition leads to a more fragmented and less transparent picture. This can be understood in terms of AO contributions to each MO. If two domain AOs contribute (almost equally) to a localized MO, the weighted CT_*w*_ will distribute the domain contributions of the CI vector component accordingly, while the hard CT measure will assign it to a single domain only. We should note, however, that this domain-assignment problem becomes negligible in larger molecules partitioned into a minimal number of domains.

Considering simpler EOM approximations, the EOM-pCCD+S results qualitatively reproduce the trends observed at the EOM-CCSD level (see also Figure S3). However, the magnitude of the CT character is systematically underestimated. Despite this quantitative discrepancy, both methods yield consistently similar qualitative behavior. This observation supports the use of EOM-pCCD+S as a more computationally efficient alternative for qualitative CT analysis, focusing on changes in the CT character upon, for instance, doping or domain modifications/substitutions. Finally, the basis set analysis shown in Figure S2 in the SI confirms that both CT descriptors are largely insensitive to the choice of basis set. The small mean errors and standard deviations indicate that the cc-pVTZ basis provides a reliable reference, and that lower-cost basis sets can be used without significantly affecting the qualitative conclusions. This is further supported by the violin plots in Figure S2 in the SI, where the distributions of the EOM-pCCD+S errors remain narrow and largely unchanged across different basis sets.

In this work, we developed and benchmarked a flexible, automated framework for analyzing CT in excited states, which is integrated into the DAISpY module of the Pythonic Black-box Electronic Structure Tool (PyBEST) quantum chemistry package.^63,64^ Two complementary domain accumulation schemes are available, namely a strict (hard) partitioning and a weighted scheme designed for smaller molecules and large domain numbers. We benchmark our approach for EOM-CCSD, various basis set sizes, and CT states (intra- and intermolecular ones). For intermolecular CT, both the excitation energies and the CT character obtained with EOM-CCSD are largely independent of the chosen reference determinant (or MO basis), including localized pCCD-optimized and canonical HF orbitals. Although the excitation energies computed with EOM-pCCD+S are generally higher by approximately 2–6 eV, the overall CT character remains very similar to EOM-CCSD results. In most intermolecular cases, the CT character exhibits a strong basis-set insensitivity, making the proposed automated domain-based CT analysis a suitable tool to efficiently and reliably resolve the excited-state character. Furthermore, both the hard and weighted domain accumulation schemes yield consistent results for the intermolecular CT. Due to its additivity with respect to domain contributions, the weighted recipe is, however, preferred, in particular for smaller molecules. Although some variations, particularly for systems such as azulene (EOM-CCSD and EOM-pCCD+S) and BTD (EOM-CCSD), are observed, the overall trends remain consistent across all investigated CT systems. Due to the basis-set independence of the intramolecular CT, already smaller basis sets can be employed to resolve domain-based excitations. Finally, in comparison to EOM-CCSD, the EOM-pCCD+S method generally yields lower CT values for intramolecular excitations, while preserving the qualitative trends observed with the former method. It thus represents a cheap alternative to dissecting excited-state characters into domain contributions, which makes it especially appealing for large-scale systems. While EOM-pCCD+S is more approximate and not intended to replace broadly available approaches such as TD-DFT to predict reliable excitation energies, it offers a computationally economical, internally consistent perspective on CT states and the evolution of the CT character upon structural modification. Diffuse basis functions remain essential for accurately describing various CT excitations. This challenge is intrinsic to the excited-state problem rather than a deficiency of the proposed methodology. The unique potential of pCCD to strongly localize virtual orbitals alleviates some of the complications introduced by very diffuse functions, although it cannot eliminate them entirely. In future work, we plan to probe the sensitivity of the DAISpY workflow on different choices of localization procedures, which might further accelerate the proposed domain-based CT decomposition, skipping the pCCD-based localization step. Furthermore, we will compare the present scheme with alternative CT analysis approaches, including density-based schemes, once excited-state densities become available in our workflow. Such comparisons will allow us to further assess the robustness and generality of the proposed methodology. Overall, the presented framework provides a robust and efficient tool for a systematic and flexible, automated, domain-based excited-state analysis. Owing to its formulation in terms of CI-type wave function amplitudes, the scheme is readily applicable to a broad range of excited-state methods, avoiding explicit left-eigenvector or excited-state density evaluations. Finally, the proposed approach can be straightforwardly extended to higher-order excitations or other excited-state models in the future.

## Computational Details

All calculations were performed using variationally optimized pCCD orbitals if not stated otherwise. In all CC calculations, a frozen core approximation was applied, keeping 1s for C, N, O, and F, and 1s–2p for S frozen. We employed two EOM variants to study the CT character and to obtain the excitation energies in the considered systems, namely EOM-CCSD and EOM-pCCD+S. All calculations were performed with the PyBEST v.2.2.0.dev0 software package,^63,64^ using GPU-acceleration for EOM-CCSD and some of the larger pCCD cases.^65^ In the case of intramolecular CT, the cc-pVDZ, cc-pVTZ, aug-cc-pVDZ, and aug-cc-pVTZ basis sets were applied.^66,67^ For intermolecular CT, only cc-pVDZ and cc-pVTZ basis sets were used. In all calculations, Cholesky-decomposed electron repulsion integrals were used with a Cholesky threshold of 10^–4^, which is sufficient to obtain excitation energies with subchemical accuracy.^68^ The intermolecular CT test set is shown in Figure 2a, while Figure 2b summarizes the intramolecular test set. The molecular structures were taken from the literature.^61,62^ DAISpY as a PyBEST interface was used for the CT analysis.^53^

## Supplementary Material





## Data Availability

The data underlying this study are available in the published article and its . The released version of the PyBEST code is available on Zenodo at https://zenodo.org/records/10069179 and on PyPI at https://pypi.org/project/pybest/. A developer, stand-alone version of DAISpY is available on GitLab at https://gitlab.com/pybest-edev/ct-analysis.

## Notes

Weighted vs hard intermolecular directed CT, additivity of the weighted CT framework; violin plots for EOM-pCCD+S, inter- and intramolecular analysis for EOM-pCCD+S (PDF)
DAISpY data (excitation energies, hard and weighted CT values) (XLSX)
