# Supplementary material for: A Flexible, Automated, and Basis-Set-Insensitive Domain-Based Charge-Transfer Decomposition for Correlated Wave Functions and Its Application to Inter- and Intramolecular Cases
Source: J Phys Chem Lett. 2026 May 22;17(22):6245–55. doi: 10.1021/acs.jpclett.6c01049 (PMC13308997; doi:10.1021/acs.jpclett.6c01049)
Supplement: Supplementary file 1 [file jz6c01049_si_001.pdf]

A Flexible, Automated, and Basis-Set Insensitive Domain-Based Charge-Transfer Decomposition for Correlated Wave Functions and its Application to Inter- and Intramolecular Cases

Lena Szczuczko,[a] Julia Szczuczko,[a] Marta Gałyńska,[b*] and Katharina Boguslawski[a*]

[a] *Institute of Physics, Faculty of Physics, Astronomy and Informatics, Nicolaus Copernicus University in Toruń, Grudziądzka 5, 87-100 Toruń, Poland*

[b] *Faculty of Chemistry, Nicolaus Copernicus University in Toruń, Gagarina 7, 87-100 Toruń, Poland*

*Email: marta.galynska@umk.pl, k.boguslawski@umk.pl

Supplementary Information

S1 Weighted vs hard intermolecular CT

	CCSD		pCCD	
molecule (A–B)	hard	weighted	hard	weighted
acetone–fluorine	0.93	0.93	0.73	0.73
acetone–nitromethane	0.68	0.68	0.57	0.59
ammonia–fluorine	0.79	0.77	0.85	0.83
ammonia–oxygendifluoride	0.61	0.61	0.89	0.89
ammonia–pyrazine	0.75	0.74	0.56	0.55
pyrazine–fluorine	0.90	0.90	0.90	0.89
	0.95	0.95		
pyrrole–pyrazine (H-bond)			0.95	0.95
			0.96	0.99
			0.96	0.96
pyrrole–pyrazine (stacked)			0.44	0.44
			0.46	0.45
			0.57	0.57
tetrafluoroethylene–ethylene (5 Å)	0.94	0.94	0.95	0.95
tetrafluoroethylene–ethylene (3.5 Å)	0.35	0.35	0.38	0.38

Table S1: Comparison of weighted and hard charge transfer for intermolecular systems for cc-pVTZ basis set. The CT shown is the % of the excitation contribution where charge flows from molecule A to molecule B.

S2 Additivity of the weighted CT framework

2-domain CT with domains “AB” and “C”		
Excitation energy [eV]	6.69	6.75
AB→AB	81.25	71.40
AB→C	12.51	22.31
C→AB	4.84	3.68
C→C	1.41	2.62
local (AB→AB + C→C)	82.66	74.02
directed CT (AB→C − C→AB)	7.67	18.63

3-domain CT with domains “A”, “B”, and “C”		
Excitation energy [eV]	6.69	6.75
A→A	32.90	28.93
A→B	32.64	28.99
B→A	7.90	6.73
B→B	7.81	6.76
A→C	9.96	17.62
B→C	2.55	4.69
C→A	2.43	1.80
C→B	2.41	1.88
C→C	1.41	2.62
local (A→A + B→B + C→C)	42.12	38.31
directed CT (A→B→C − C→B→A)	32.41	40.89

2-domain reconstructed from 3-domain CT with domains “A+B” and “C”		
Excitation energy [eV]	6.69	6.75
A→A + B→B + A→B + B→A	81.25	71.41
A→C + B→C	12.51	22.31
C→A + C→B	4.84	3.68
C→C	1.41	2.62
local ([A+B]→[A+B] + C→C)	82.66	74.03
directed CT ([A+B]→C − C→[A+B])	7.67	18.63

Table S2: Additivity of the weighted charge transfer (CT_w) for phtalazine computed with EOM-pCCD+S/cc-pVDZ. CT results [%] are shown for a two-domain partitioning (top left), a three-domain partitioning (top right), and the reconstructed two-domain quantities obtained by summing the corresponding three-domain contributions (bottom). The 2- and 3-domain partitionings are shown in Figure S1. The agreement between the direct two-domain results (“AB” and “C” case) and the reconstructed values (for “A”, “B”, and “C”) demonstrates the additivity of CT_w.

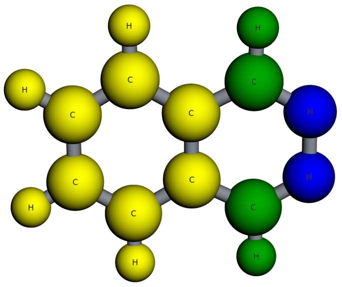

Figure S1: 3-domain partitioning of phtalazine. Colours indicate molecular roles: blue for the donor (“A”), green for the linker (“B”), and yellow for the acceptor (“C”). The visualisation was generated using 3Dmol via DAISpY’s graphical user interface.

2-domain CT with domains “AB” and “C”		
Excitation energy [eV]	6.69	6.75
AB→AB	75.5	73.59
AB→C	16.74	17.75
C→AB	7.35	5.26
C→C	1.41	3.40
local (AB→AB + C→C)	76.91	76.99
directed CT (AB→C − C→AB)	9.39	12.49

3-domain CT with domains “A”, “B”, and “C”		
Excitation energy [eV]	6.69	6.75
A→A	80.95	70.86
A→B	3.00	1.77
B→A	0.52	0.91
B→B	0.03	0.05
A→C	6.57	17.63
B→C	0.17	0.12
C→A	7.04	5.05
C→B	0.31	0.21
C→C	1.41	3.40
local (A→A + B→B + C→C)	82.39	74.31
directed CT (A→B→C − C→B→A)	1.87	13.35

2-domain reconstructed from 3-domain CT with domains “A+B” and “C”		
Excitation energy [eV]	6.69	6.75
A→A + B→B + A→B + B→A	84.50	73.59
A→C + B→C	6.74	17.75
C→A + C→B	7.35	5.26
C→C	1.41	3.40
local ([A+B]→[A+B] + C→C)	85.91	76.99
directed CT ([A+B]→C − C→[A+B])	-0.61	12.49

Table S3: Additivity (or lackthereof) of the hard charge transfer (CT) for phtalazine computed with EOM-pCCD+S/cc-pVDZ. CT results [%] are shown for a two-domain partitioning (top left), a three-domain partitioning (top right), and the reconstructed two-domain quantities obtained by summing the corresponding three-domain contributions (bottom). The 2- and 3-domain partitionings are shown in Figure S1 and Figure 1 in the main text. Comparison of the direct two-domain results (“AB” and “C”) with the reconstructed values (“A”, “B”, “C”) illustrates that the hard scheme can exhibit additivity for some states (e.g., the second state) but not for others (e.g., the first state).

S3 Violin plots for EOM-pCCD+S

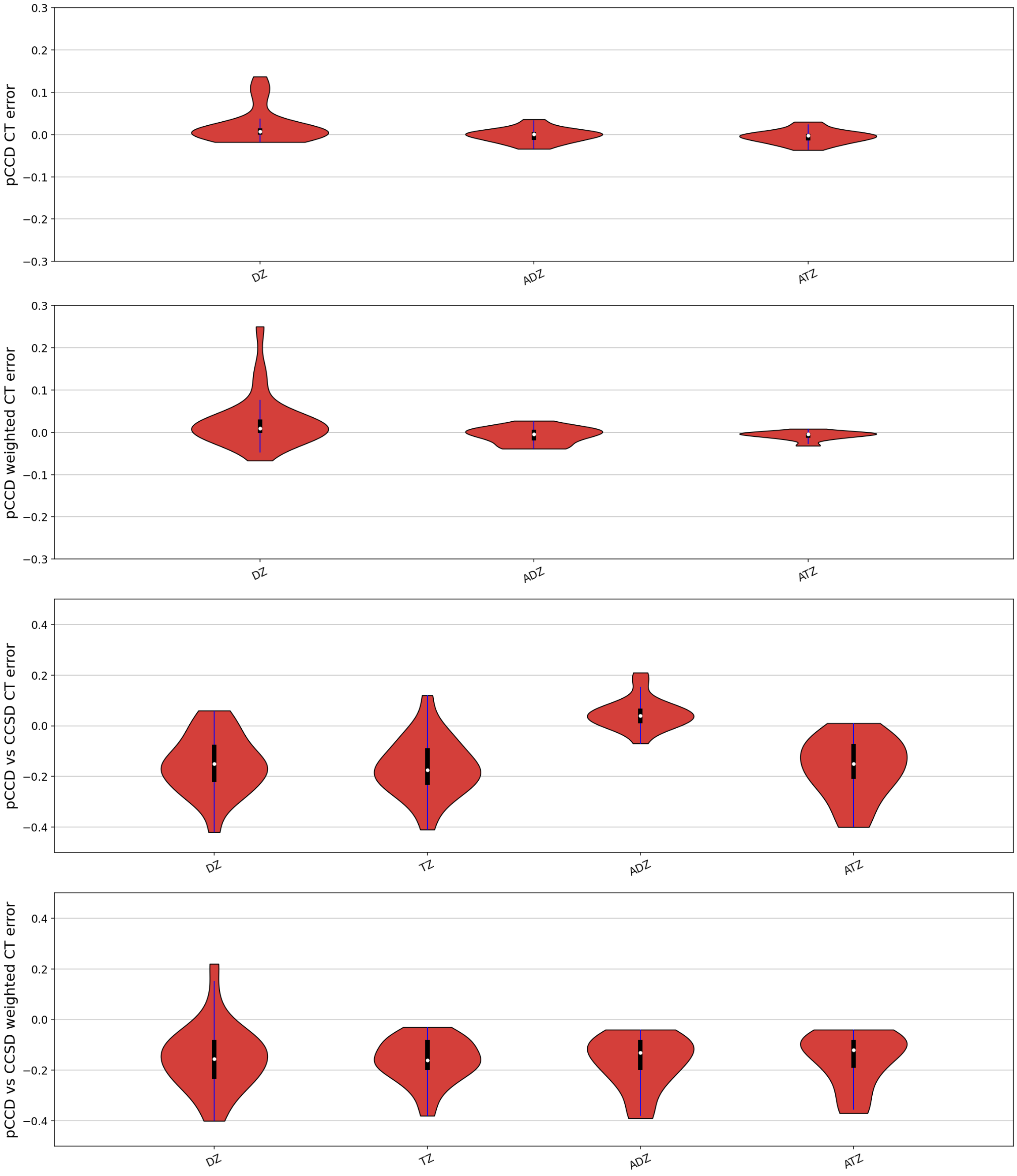

Figure S2: Violin plots of the errors in the hard and weighted EOM-pCCD+S dCT values. The upper panels show the deviation of EOM-pCCD+S results obtained with the cc-pVDZ, aug-cc-pVDZ, and aug-cc-pVTZ basis sets with respect to the cc-pVTZ reference. The lower panels compare the errors between EOM-pCCD+S and EOM-CCSD across all basis sets. Results are shown separately for the hard and weighted dCT.

S4 Intermolecular analysis for EOM-pCCD+S

		CCSD(HF)[?]		CCSD(pCCD)		pCCD+S(pCCD)	
Molecule	Basis set	EE	ω_{CT}	EE	CT	EE	CT
acetone–fluorine	DZ	6.28	0.96	6.33	0.94	11.04	0.39
	TZ			6.02	0.93	11.50	0.73
acetone–nitromethane	DZ	6.75	0.80	6.84	0.69	10.68	0.80
	TZ			6.64	0.68	10.31	0.57
ammonia–fluorine	DZ	6.90	0.76	6.87	0.79	11.12	0.84
	TZ			6.63	0.79	11.21	0.85
ammonia–oxygendifluoride	DZ	7.33	0.86	7.37	0.84	12.12	0.82
	TZ			7.33	0.61	12.38	0.89
ammonia–pyrazine	DZ	7.93	0.63	7.95	0.61	10.65	0.64
	TZ			7.81	0.75	10.59	0.56
pyrazine–fluorine	DZ	6.73	0.64	6.74	0.64	9.93	0.94
		6.77	0.98	6.77	0.94	11.74	0.99
	TZ			6.28	0.90	9.74	0.90
				6.37	0.95		
pyrrole–pyrazine (H-bond)	DZ	5.60	1.00	5.64	0.96	8.04	0.95
		6.32	0.97	6.32	0.92	8.91	1.00
		6.47	0.99	6.51	0.95	9.00	0.98
	TZ					7.90	0.95
						8.82	0.96
						8.89	0.96
pyrrole–pyrazine (stacked)	DZ	5.68	0.84	5.69	0.81	8.25	0.57
		6.22	0.66	6.24	0.62	8.30	0.41
		6.52	0.61	6.51	0.58	9.34	0.76
	TZ					8.02	0.44
						8.09	0.46
						9.22	0.57
tetrafluoroethylene–ethylene (5 Å)	DZ	10.87	0.99	10.87	0.97	13.75	0.99
	TZ			10.54	0.94	13.22	0.95
tetrafluoroethylene–ethylene (3.5 Å)	DZ	9.05	0.34	9.08	0.36	11.48	0.29
	TZ			8.66	0.35	10.95	0.38

Table S4: Intermolecular CT character (all CT values are of 1→2 character, i.e., from the first fragment listed in the molecular name to the second) and excitation energies (EE in eV) calculated with cc-pVDZ (DZ) and cc-pVTZ (TZ) basis sets using EOM-pCCD+S, EOM-CCSD(pCCD), and reference EOM/LR-CCSD(HF) data. Missing values have not been computed. (HF): indicates that canonical Hartree–Fock orbitals are used to construct the reference determinant. (pCCD): indicates a reference determinant constructed with pCCD-optimized orbitals.

S5 Intramolecular analysis for EOM-pCCD+S

		EOM-CCSD			EOM-pCCD+S		
molecule	dCT char.	EE	dCT	dCT$_w$	EE	dCT	dCT$_w$
ABN	++	5.41	0.29	0.23	6.45	0.16	0.10
aniline	++	5.99	0.23	0.18	6.78	0.09	0.06
benzonitrile	++	7.33	0.27	0.27	8.37	0.08	0.10
DMAn 1	++	4.66	0.21	0.18	5.78	0.07	0.05
DMAn 2	++	5.68	0.36	0.29	6.68	0.12	0.09
nitrobenzene	+++	5.77	0.53	0.48	6.64	0.25	0.23
NPNO	++	4.45	0.31	0.29	5.49	0.14	0.13
azulene 1	++	4.03	0.41	0.20	4.80	0.34	0.13
azulene 2	+	4.83	0.24	0.10	6.27	0.19	0.03
BTD	++	4.67	0.34	0.19	5.75	0.19	0.11
DMABN	++	5.10	0.40	0.35	6.35	0.19	0.15
pNA	+++	4.81	0.50	0.44	6.23	0.31	0.26
nDMAN	+++	4.53	0.62	0.56	6.09	0.30	0.24
PP 1	+++	5.87	0.42	0.41	7.18	0.15	0.15
PP 2	++++	6.56	0.75	0.74	9.21	0.51	0.50
phtalazine 1	++	4.28	0.09	0.17	6.84	0.09	0.09
phtalazine 2	++	4.64	0.30	0.34	6.89	0.12	0.19
quinoxaline 1	+++	5.04	0.59	0.54	6.37	0.18	0.16
quinoxaline 2	++	5.98	0.34	0.30	7.07	0.32	0.27
twisted DMABN 1	++++	4.42	0.84	0.77	7.21	0.79	0.71
twisted DMABN 2	++++	5.19	0.83	0.76	8.42	0.75	0.69
twisted PP 1	++++	6.13	0.87	0.85	8.46	0.68	0.66
twisted PP 2	+++	6.22	0.62	0.62	8.55	0.74	0.69
twisted PP 3	++++	6.33	0.80	0.75	9.02	0.55	0.55
ME (DZ)	–	–	0.01	0.02	–	0.02	0.03
SD (DZ)	–	–	0.06	0.07	–	0.04	0.06
ME (ADZ)	–	–	-0.03	-0.02	–	0.00	-0.01
SD (ADZ)	–	–	0.10	0.10	–	0.02	0.02
ME (ATZ)	–	–	-0.02	-0.04	–	0.00	-0.01
SD (ATZ)	–	–	0.06	0.06	–	0.02	0.01

Table S5: Intramolecular hard (dCT) and weighted (dCT$_w$) dCT character and excitation energies (EE in eV) calculated with the cc-pVTZ basis set using EOM-pCCD+S and EOM-CCSD. For some molecules, more than one excited state is reported. The qualitative dCT character (dCT char.) is assigned based on the weighted EOM-CCSD dCT$_w$ values: weak (+) for $|\text{dCT}_w| \leq 0.10$, moderate (++) for $0.10 < |\text{dCT}_w| \leq 0.35$, strong (+++) for $0.35 < |\text{dCT}_w| \leq 0.7$, and (mostly) pure (++++) for $0.7 < |\text{dCT}_w| \leq 1.0$. Mean error (ME) and standard deviation (SD) are also provided for the cc-pVDZ (DZ), aug-cc-pVDZ (ADZ), and aug-cc-pVTZ (ATZ) basis sets, using the cc-pVTZ results as reference values. The distribution of these statistics is illustrated by the violin plots shown in Figure S2 in the SI.

S6 Comparison of intramolecular analysis for EOM-pCCD+S and EOM-CCSD

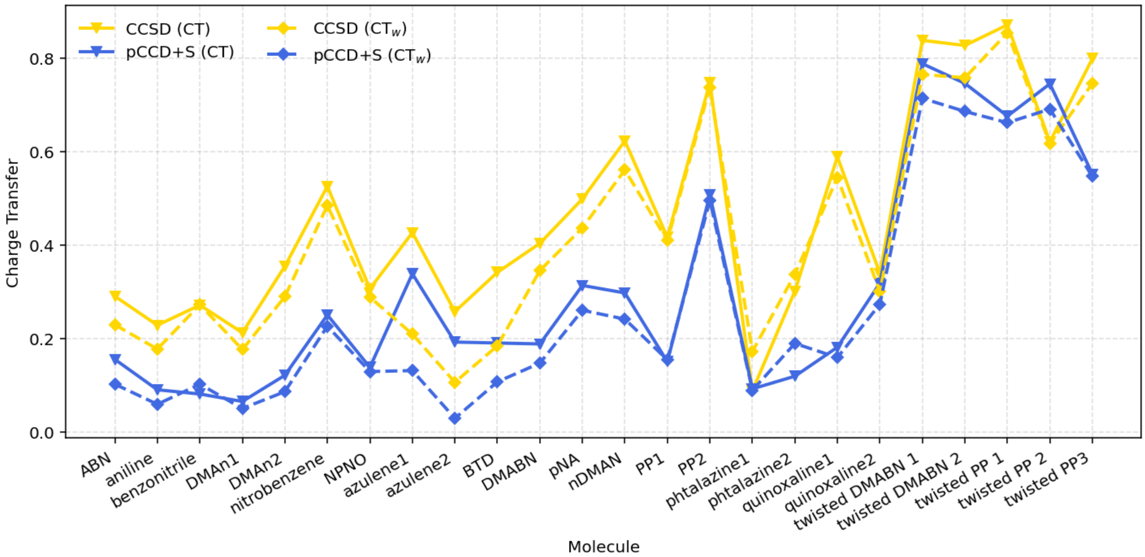

Figure S3: Comparison of EOM-CCSD and EOM-pCCD+S results for strict and weighted dCT character.
